# From strict moral standards to ethical neutrality: a policy-guided shift in the patentability of human embryonic stem cells in China

**DOI:** 10.1186/s13287-020-02013-x

**Published:** 2020-11-25

**Authors:** Xuekai Xie, Jiajv Chen, Zhengyang Shu

**Affiliations:** 1 School of Law, East China University of Science and Technology, 130 Meilong Road, Xuhui District, Shanghai, China; 2 School of Law, Zhejiang University of Finance & Economics, Hangzhou, 18 Xueyuan Street, Jianggan District, Hangzhou, Zhejiang China

**Keywords:** Human embryonic stem cells (hESCs), Ethics, Policy, Patentability

## Abstract

Attitudes towards human embryonic stem cells (hESCs) in China have witnessed a significant shift in 2020 that can be attributed to China’s policy guidance. For ethical reasons, stricter standards are adopted to curb related regulations and patent licensing. Through the introduction of policies, some research on hESCs has been recognized as legitimate and feasible to a certain standard and scope. In the subsequent practice of patent examination, the dual influence of policy support and public interest has led to a shift in the examination standards of China’s intellectual property authority from “strict morality” to “ethical neutrality”, implying limited recognition of hESCs’ patentability. In view of the promotion of policy incentives for the transformation and application of corresponding research, there is considerable social demand to provide patent protection for research results. In this context, an adjustment of related regulations is illustrated in this revision, manifesting a partial shift in regulations towards a supportive stance consistent with policy.

## Introduction

In terms of both resources and the quality of research on stem cells, China’s bank of human embryonic stem cells (hESCs) is at the forefront internationally. As of June 2019, 9292 patent applications for hESCs have been filed in China, ranking the country first in the world^1^. Nevertheless, due to China’s rigorous regulations, only 246 have been effectively licensed^2^. The patentability of hESCs is mainly covered by three progressive regulations, namely, the *Chinese Patent Law* (CPL), *Implementing Regulations of the Patent Law of the People*’*s Republic of China* and the *Guidelines for Patent Examination* (GPE). As a specific standard for patent applications and requests in accordance with the other two regulations, the GPE are crucial in the practice of patent review. The patentability of an invention “contrary to social morality” is excluded in article 5 of the CPL^3^ despite the licensing of a human embryo not being explicitly provided for. In the early GPE (2006), it was further stipulated that “the application of human embryonics for industrial or commercial purposes” shall be deemed a violation of social morality, that “hESCs and their preparation are not patentable objects”, and that “germ cells, fertilized eggs, embryos and individuals shall not be granted patent rights as stages of human formation and development”, suggesting great limitations placed on the licensing of inventions related to human embryos. However, with the great potential and value of human embryo technology recognized internationally, especially in the field of medicine [1], corresponding protection mechanisms have been voluntarily studied and constructed by various countries. After the USA, patent protection as one of the most effective models is recognized in the European Union, the UK, and Japan through judicial practice, revealing the inevitability of patent protection for human embryo research.

Chinese policy plays a positive role in promoting patent protection for hESCs. Regulation of the patentability of human embryos in China was initially rejected on ethical grounds. One example is the case of one Japanese citizen, Tsuneo Kido, whose invention involving hESCs was quickly granted a patent license in countries including the USA and Japan and was later approved by the European Patent Office. In contrast, it was rejected by the Patent Reexamination Board of CNIPA (CPRB) and the Beijing Intellectual Property Court as an “application for industrial or commercial uses of human embryos” [2], reflecting China’s stringent standards. However, with the growing maturity and emerging value of human embryonic technology, different opinions have been raised about the patentability of hESCs. Xiuyun expressed concern about China’s policy of strictly excluding the patentability of hESCs and believed that this would have adverse effects on scientific research and public health [3]. According to Qiang, patents were by no means the only way or the last line of defense to safeguard ethics, as the impact of technological development on ethics can actually be offset by multiple approaches such as administrative law. The overly abstract definition of a human embryo used in China is bound to impede the development of technology [4], boosting calls for the patentability of human embryos. In as early as 2001, the Chinese government issued the *Ethical Guidelines for hESC Research* (*draft*). Apart from the elaboration of ethical and moral hazards of human embryos and the setting of a series of restrictions, a basic policy of encouraging and supporting limited research on hESCs was formulated. In this way, China’s hESC research has been justified and promoted. In the following years, a number of normative documents were issued in China to guide and encourage the further development of hESCs, which has had a positive impact on China’s patent examination practices. With recognition of the patentability of hESCs, the review rules were well established and reflected in the revision of the GPE. Based on the revision of the GPE, we attempt to reveal the impact of China’s relevant policies on social ethical and regulation changes regarding the patentability of human embryos and to explore the content of the revision.

## Policy guidance under ethical restraints

### Ethical restraints

Located in the inner layer of the blastocyst, hESCs are a group of self-renewing pluripotent cells that, under the right conditions, can differentiate into nuclear tissues of the human body and eventually develop into organs. In biology, an embryo can develop into a human being, though not in the full sense of the word. According to Western religious thinking, the embryo formed at the moment the sperm and egg meet constitutes life and must be protected. The embryo is taken as the embryonic form of a human being and given the same protection, so its research and utilization is prohibited. According to the Confucian theory of gradualism, a human being first exists at birth rather than with the formation of a fertilized egg. Given the influence of Confucianism on Chinese society, human embryos and fetuses are not considered equivalent to human beings [5]. Despite the differences between Chinese and Western views of human origins, this does not mean that China does not agree with the ethics of embryos. As a basic principle of Chinese civil law, public order and good customs are embodiments of ethics in law. In China, ethical concerns about embryos mainly emerge in areas of human cloning and the destruction of human genetic consistency. Hence, ethical and moral restrictions placed on human embryos in China have restrained the regulation and practice of hESC patentability [6].

In regulations, strict EU regulations were adopted [7]. In China’s early practice, the “patentability of excluding the utilization of human embryos for industrial or commercial purposes” in the EU’s biotechnology patent directive was followed, yet the “patentability of inventions that are allowed to be applied to human embryos for the purpose of treatment or diagnosis and are beneficial to human embryos” was not exempted as in the EU with more stringent ethical requirements being put forward.^4^ In patent examination, concepts of the human embryo and of embryonic stem cells failed to be clearly defined under China’s regulations. Due to ethical pressures and implications, the State Intellectual Property Office of CNIPA (CSIPO) resorted to expansive interpretations on most occasions. In early practice, the source of hESCs was traced to the destruction of embryos, leading to the majority of applications being rejected at that time for a breach of public order and good manners.

### Policy guidance

In view of the different understanding of human origins, the possibility of carrying out research on human embryos has never been completely excluded in Chinese society. The China National Human Genome South Research Center (CHGC) was founded in October 1998 and issued its *draft* in 2001. Through the introduction of policies, the legitimacy of research on hESCs under certain conditions was specified. Although the results could not yet be protected by a patent, the legitimacy and feasibility of some research was established, reserving space for the development of stem cell technology in China. A number of guidelines were issued in subsequent years, with five issued in 2003 alone. Among them, the former Ministry of Health (now renamed the National Health and Family Planning Commission of the PRC) successively promulgated the *Norms of Human Assisted Reproductive Technology*, *Ethical Principles for the Implementation of Human Assisted Reproductive Technology* and *Ethical Examination Methods for Biomedical Research Involving Human Beings*. Apart from clarifying the basic principles and scope of research on hESCs and ways to obtain hESCs, relevant inspection and supervision measures were detailed. In the *Guiding Principles of Human Gene Therapy Research and Preparation Quality Control Technology* and *Guiding Principles of Human Cell Therapy Research and Preparation Quality Control Technology* issued by the National Medical Products Administration, hESC research in China was standardized by prescribing and clarifying the clinical study of gene editing involving hESCs. In addition, according to the *National Science and Technology Innovation Plan of the 13th Five*-*year Plan* and the *Notice on the Release of 2017 Annual Project Application Guide for National Key Research and Development Projects for hESCs and Translational Research* issued by the State Council and Ministry of Science and Technology, colleges, universities, and scientific research institutions were encouraged to apply for hESC research projects with funds provided as an effective approach to the mobilization of domestic scientific research. Benefitting from favorable policies, in addition to scientific research institutions including the National hESCs Engineering Technology Research Center and National Engineering Research Center for hESCs, major Chinese universities such as Tsinghua University and Peking University established cell research centers of their own. In contrast to Europe’s decreased investment in biotechnology in recent years, especially in agricultural biotechnology due to great opposition to transgenic technology, investment in China has increased, which has been inseparable from policy guidance [8]. With the development of basic clinical research on hESCs, plant cell totipotency and organogenesis as a result of policy support, China has witnessed great progress in hESC technologies [9].

## Evolution of patent examination standards under policy incentives

### Policy incentives

Since confirming their legitimacy and standardization, research on hESCs has been listed as a national key development project since 2005. According to the *Outline of National Medium and Long*-*term Science and Technology Development Plan* (*2006–2020*), “hESC-based human tissue engineering technology” was expected to be the key research focus of cutting-edge technology over the next 15 years [10], governing the direction and pattern of China’s future development of biotechnology. In 2010, hESC-related studies were included as key projects in China’s national science and technology programs “863” and “973”, marking the full development of research on hESCs in China. According to the “*863*” *Program Application Guide for Biological and Pharmaceutical Technology Projects* issued by the China Biotechnology Development Center of the Ministry of Science and Technology in December 2010, it was stipulated that the clinical translation and applied research of hESC therapy technology and the development of digital medical engineering technology was to be funded as theme projects. Among the nine major scientific issues examined in the “973” program identified in the *12th Five*-*year Plan of National Major Scientific Research Plan for hESC Research* issued in 2012, hESC issues accounted for three, making it the most approved research area. As a result of policy incentives, China’s hESC research has achieved fruitful results with extensive application. At the forefront of the world in basic research on hESCs, transplantation, and clinical applications of cord blood, China is playing a crucial role [11]. Moreover, research on hESC therapy for spinal cord injury, stroke, brain injury, and cerebral palsy has made considerable progress [12]. As the research results reveal, hESCs’ significant gains in public interest have been gradually recognized, redefining the balance between ethics and public interest [13]. Due to the influence of policies and public interest, China’s patent examination standards demonstrate a shift from strict morality to ethical neutrality in this period.

### Changes in patent examination rules

#### Restrictions under strict moral standards

Before the revision of 2020, section 9.1.1.1 of chapter 10, part II of the GPE stipulated that “hESCs and their preparation methods are inventions that cannot be patented according to paragraph 1 of article 5 of the CPL”, while section 9.1.1.2 stipulated that “human bodies at all stages of formation and development (including human germ cells, fertilized eggs, embryos and individuals) are inventions that cannot be patented according to paragraph 1 of article 5 of the CPL” [14]. These are embodiments of the “strict ethical standards” China adopted in its examination of hESCs. Not only is there no patent granted for the “application of the human embryo industry or commercial purposes”, the embryonic stem cells themselves and their preparation methods are also expressly prohibited. The “embryonic or stem-like cell lines produced by cross species nuclear transplantation (Application No.: 97198083.7)” applied by the University of Massachusetts in 1997 and the “method for separating and cultivating human multifunctional embryo stem cells (Application No.: 03826612.1)” applied by Lu Guangxiu’s team from China in 2003 were both rejected because the stem cell source involved in the claims and specifications involved human embryos. Nevertheless, there was no provision in the regulation for granting patents for “indirect” inventions such as the differentiation, use and preservation of hESCs, which might be patented in earlier applications. An example is the “human embryonic stem cell methods and podxl expression (Application No.: CN200780008235.3)” applied for authorization in 2007, involving “a method of identifying an undifferentiated human embryonic stem cell in a sample which may contain such cells, the method comprising identifying the cell or cells within the sample that express podocalyxin-like protein (PODXL) on their surface; a method of isolating an undifferentiated human embryonic stem cell from a sample containing such cells; and the method involving isolating the cell or cells within the sample that express PODXL on their surface”^5^, which was eventually authorized. In addition, the method of hESC preservation might also obtain patent authorization as evidenced by law on the “low-toxicity vitrified frozen solution of human embryonic stem cells (hESCs) and method of using the same (Application No.: CN201010266751. X)” applied in 2010. This was authorized for revealing the vitrified cryopreservation of hESCs with low toxicity.

The rationality of early patent examination rules was challenged by multiple parties. Some scholars asked if human embryos and their preparation methods were taken as immoral and nonpatentable out of respect for ethics, were the techniques derived from such inventions such as extraction, preservation, and the promotion of differentiation ethical? That is, are the fruits of a poisonous tree edible? [15] Inspired by this thought, there was more reflection on the “strict ethical standards” imposed in China. China’s practitioners were equally critical of this. It was believed to be a violation of the upper law, as the CPL did not rule out the possibility of hESCs being patented. The licensing rules for hESCs were expected to be clarified to inhibit expansive interpretations of related concepts.

#### Gradual relaxation under ethical neutrality

The application of the rule of “strict moral standards” has led to a debate regarding whether hESC-related inventions conform to social ethics. According to advocators, the CPL is to encourage technological innovation and promote social progress, and since technological inventions are not inherently subjective, whether patents are granted will not ensure the elimination of undesired technology development and implementation [16]. In contrast, while acknowledging the objectivity and neutrality of technology, advocators of ethical judgment argue that the choice and use of the technology depends on people’s subjective preferences, implying that patent protection through regulation will encourage human embryo research. The CPL is not expected to provide incentives for acts that violate laws or good customs, though it cannot prevent such acts [17].

The public order and good custom provisions stipulated in the CPL reflect a moral examination of patent applications, which is also the fundamental principle on which the patentability of human embryos is excluded. In spite of the neutrality of technology, the normative guidance of law in directing human behavior should be legitimate and moral and therefore should not completely rule out the ethical test of patents. From the perspective of patent examination, instead of a uniform definition, moral standards are entirely based on the subjective judgments of examiners, which will inevitably lead to the improper expansion of their coverage. Therefore, it is necessary to include limited ethical review during examination to avoid expansive interpretations. Moreover, for the maturity of human embryonic technologies in China, the number of patent applications has soared, prompting calls for patent protection for related technologies. In this context, from the initial “strict moral standards” to those of “ethical neutrality”, the CPRB began to offer limited recognition of the patentability of hESCs through a series of reexamination decisions that relaxed the standards. The shift is mainly reflected in (1) the differentiation of stem cell types and (2) a lack of tracing of hESC sources.

### Classification of hESC types

Relevant biological studies have shown that only totipotent stem cells have the potential to develop into human beings [18]. Some countries such as the UK have made it clear that totipotent stem cells cannot be patented [19]. However, for nontotipotent stem cells such as pluripotent stem cells obtained from human embryonic stem cells by modification or other means, patents can be granted as long as they comply with other provisions of the British patent law [20]. Although human embryos are not defined in China, they are not distinguished in patent examination practice and are in most cases interpreted as a process of human development to exclude their patentability [21]. In its examination decision on “haematopoietic cells from human embryonic stem cells (Application No.: CN:02827064:A)”, the CPRB held that since the “undifferentiated human embryonic stem cell population” can differentiate and develop into a complete human being by virtue of its totipotent differentiation, it should be regarded as a human body at various stages of formation and development, which is one of the nonpatentable inventions stipulated in article 5 of the CPL^6^. For the definition of “human embryos”, in its examination decision of the “method for observing parthenogenetic embryonic stem cells (Application No.: CN:201010266776:A)”, broad interpretation was adopted by the CPRB. This was believed to not be limited to the development of human embryos in their natural state but to also extend to unfertilized and immature forms. Therefore, even when its access did not involve the destruction of stem cells, it might still not be authorized^7^. This extremely narrow determination was reversed in the 2015 review of the “nuclear reprogramming factor (Application No.: CN:200680048227:A)”. According to the CPRB, since human cells without developmental totipotency were not in the category of human embryos, the invention of biological methods based on them did not constitute an ethical violation. Thus, the patentability of some hESCs was affirmed, while commercially derived stem cells were not deemed directly from human embryos in violation of article 5 of the CPL^8^. The decision reflected a limited recognition of the patentability of certain stem cells, reflecting a shift in China’s stance on the patentability of hESCs.

### Justification of the origins of commercial hESCs

Obtaining patent authorization for hESCs in China is a challenge in that they usually originate directly from human embryos, thus failing to be taken as patent objects involving social ethics. Nevertheless, hESCs may also survive and reproduce in vitro. Thomson of the University of Wisconsin established five hESC lines (i.e., H1, H7, H13, h14, and H9) in as early as 1998 that could be passed on stably in vitro [22]. With the establishment of a growing number of hESC lines, a stable business chain was formed to enhance the possibility of related stem cell supplies. Such commercial hESCs were initially denied authorization in China. The “method of making embryoid bodies from primate embryonic stem cells (Application No.: CN: 01805291: A)” initiated by the Wisconsin Alumni Research Foundation in 2001 was rejected. After in-depth investigation, the CPRB argued that even though the applicant had indicated in claims and applications that the hESCs cultivated were from existing commercial hESC lines, their ultimate source was still human embryos. Moreover, technical conditions at the time implied that such extraction would inevitably cause damage to the embryos, so the decision to reject the application was finally upheld^9^. The retrospective examination standard for the origins of hESCs remained unchanged until the review decision of the “method for inducing human embryo stem cell differentiation to liver cell and the special-purpose medium” was released in 2010. According to the Patent Application Department, “while hESCs referred to in the claim could be obtained through commercial channels, the commercial sale of hESCs is still fundamentally based on the destruction of human embryos and thereby should not be authorized”. However, the mode of “retroactive determination” was reversed in the review. The CPRB stated that if there was evidence that the induced differentiation method did not necessarily involve the destruction of human embryos, it would not be unethical to use human embryos for industrial or commercial purposes^10^. Thus, the legitimacy of obtaining hESCs through commercial channels was recognized for the first time, marking a shift of the position on existing commercial hESCs. In the subsequent review decisions on cardiomyocyte precursors from human embryonic stem cells, oligodendrocytes derived from human embryonic stem cells for remyelination and the treatment of spinal cord injury, the differentiation of human embryonic stem cells to the pancreatic endocrine lineage, etc.^11^, the introduction of commercial stem cells was acknowledged as a sign of the growing neutrality of ethical attitudes towards hESC applications in China.

The above decisions demonstrate a shift in China’s standards for the examination of human embryo research followed by the establishment of determination rules. As China’s legislative system was a civil law system, however, the above cases were only for reference rather than shaping review practice. Hence, these rules were not fully complied with, and the review standards continued to be repeated and changed in subsequent practice, posing challenges to both reviewers and applicants. This has rendered the revision of regulations to clarify relevant rules a top priority in China’s examination practice.

## A shift in regulation towards policy support

### Coordinated regression of law and policy

Patent protection for the intellectual results of hESC research is inconsistent relative to China’s historically active policy. On the one hand, China has expended considerable manpower and financial resources to introduce policies that encourage hESC research and has achieved positive results. On the other hand, the challenges of such efforts in obtaining patent protection led to a clash of policy and law. The coordinated interaction of policy and law forms the premise of the rapid and stable development of a country. In terms of actual domestic demands, policies are more timely and flexible than laws and play a leading and guiding role. The disconnect between policy and law demonstrates a lag in China’s regulations on hESCs [23]. With the globalization of human embryo research, progress in technological development and changes in social concepts, the translation, and application of hESC-related research have been promoted in China. In the first half of 2019 alone, more than 10 documents were issued by the Ministry of Science and Technology, the National Health Commission and provinces and cities [24]. Among them, according to the *Notice on the Release of the 2017 Annual Project Application Guide for National Key Research and Development Projects for hESCs and Translational Research* issued on January 21, 2019, “in addition to the 98 projects already supported, the government will allocate another 400 million Yuan in 2019 for 12 stem cell research projects” [25] as a sign of China’s determination to continue the research. In the face of the translation and application of relevant research, their protection has become a social imperative. The revision of the GPE reflects a positive response to demand.

### Interpretation of the revision

In the *Revised Draft of the Patent Examination Guide* (*Exposure Draft*) issued in April 2019, the CSIPO clarified reasons for the revision of provisions on hESCs. “With the rapid development of *hESC* technology in recent years, patent protection requirements for the technology have been put forward by some innovative subjects. To cater to this trend, on the basis of investigating restrictions of relevant domestic laws and regulations and referring to measures of foreign patent offices, it is proposed that through the revision of the GPE, the patent protection of ‘human embryo separation or stem cell technology acquisition within 14 days of fertilization without in vivo development’ shall not be completely excluded on account of article 5 of the CPL. At the same time, section 9.1.1.1 of Chapter 10, Part II of the current edition (hESCs and their preparation methods are inventions that cannot be patented under paragraph 1, Article 5 of the CPL) was deleted, and it shall be specified in section 9.1.1.2 that ‘hESCs are not human bodies at various stages of formation and development’” [26].

In view of relevant regulations, the revision of the GPE might be summarized into two points. The first concerns the addition of an exclusion clause in section 3.1.2 of chapter 1, part II to “the application of human embryos for industrial or commercial purposes”. It is clarified that if an invention or creation employs a human embryo within 14 days of fertilization that has not been developed in vivo to separate or obtain stem cells, it should not be denied a patent on the grounds of “violating social morality”, which confirms the legitimacy of obtaining hESCs under certain conditions. The second concerns the deletion and modification of chapter 10, part II of the GPE. hESCs are defined as not reflecting stages of human development, while hESCs and their preparation methods are likely to obtain patent authorization. Overall, in taking into account factors including existing technologies and foreign backgrounds, the revision is significant for its expansion of space and opportunity for the patentability of human embryos.

### Significance and limitations

Challenges in licensing hESC inventions in China can be attributed to the fact that some basic concepts such as “application for industrial or commercial purposes” and the scope of the “human embryo” have not been clarified in previous regulations. This has led to an improper expansion of these concepts and to strict examination standards adopted in patent examination practice. Although the meaning of “application for industrial or commercial purposes” is not clearly defined, the justification for obtaining hESCs under certain conditions through the provision of exceptions is recognized in the revision. Thus, established lines of hESCs obtained from commercial sources may also meet the requirements of public order and good customs, indicating the embodiment of examination rules and the elimination of improper tracing of hESCs by patent examination authorities. Second, there is a shift in the GPE’s position on hESCs and their preparation. Instead of the previous negative legislative approach, the extensibility of relevant regulations is retained to cope with the impact of future technological development, leaving room for China’s future division of hESCs and patentability under new technology modes. Moreover, China’s policies and regulations on hESCs have long been in direct conflict. “The legitimacy of the use of the in vitro culture period from fertilization or nuclear transplantation to no more than 14 days of hESCs research” is clarified in as early as in the *Ethical Guidelines for hESCs Research* issued by the former Ministry of Health in 2003, which has been completely denied by relevant regulations in China. However, as a reversal of previous denials of the patentability of hESCs, the revision is expected to substantially promote the development of biotechnology in China by responding to and coordinating with policy support.

The GPE, on the other hand, has been revised to promote research on hESCs. There are limitations and deficiencies in view of the revised patentability failing to cover animal embryonic stem cells [27]. According to section 4 of paragraph 1, article 25 of the CPL, “animal and plant varieties” are not patentable. In section 4.4 of chapter 1 and section 9.1.2.3 of chapter 9, part II of the GPE, it is stipulated that “animals mentioned in the CPL do not include human beings” and “the embryonic stem cells of related animals are animal species”, indicating a rejection of the patentability of animal embryonic stem cells. A peculiar phenomenon thus occurs. While hESCs are patentable in China under certain conditions, animal embryonic stem cells cannot be licensed. As humans are biologically animals and there is considerable research on animal embryonic stem cells, a relaxation of the ethics of human embryos should be accompanied by a refinement of the animal embryo research regime, which might be achieved through future revisions to the CPL.

## Conclusion

In terms of the disposal of the patentability of human embryos, China’s practice is of reference significance. Rather than changing regulations to deny the ethics of human embryos or forcing a shift of stance through policies, the guiding role of policy was taken to facilitate the development of hESC technology in China. This, in turn, has led to regulative amendments. With early policy support, studies were carried out to make hESC research possible in China. In this way, ethical arguments remained focused on licensing rather than enforcement, establishing China’s leading role in related biotechnologies. With the subsequent introduction of a series of policies, the value of hESC technology became increasingly prominent, followed by a reexamination of the balance between ethics and the public interest. Due to the multiple influences of policies and public interests, the strict moral standards of patent examination practice were gradually replaced by ethical neutrality with some research results being patented. Finally, with hESC research listed as a national strategy, the translation and application of related research results began to receive policy support, leading to the era’s demand for results protection and the inevitability of amending regulations for patent protection. In the area of gene editing, benefiting in large part from China’s policy guidance and support, the China Intellectual Property Administration surpassed the European Patent Office in the number of patents granted in 2005 and the US Patent and Trademark Office in 2011 [28]. The balancing of ethics and social development in China has been maintained through a series of policy announcements that, by nurturing the country’s development of hESC technology, have contributed to China’s take-off in the field of biotechnology.

## Data Availability

The Chinese patent application information (please refer to the label in the article for the specific application number) involved in the article can be searched in the “Patent Search” section of the official website of the State Intellectual Property Office of China; website, http://www.cnipa.gov.cn/, last visit date 27 October 2020. The full text of the patent reexamination resolution involved can be checked in the “Decision Inquiry” of the Patent Office Reexamination and Invalidation Department of the State Intellectual Property Office of China (please refer to the label in the article for the specific application number); website, http://reexam.cnipa.gov.cn/, last visit date 27 October 2020. Regarding the relevant laws and regulations in China, the important content and specific legal provisions have been annotated in the article. If you want to view the full text, please log in to the Ministry of Justice of the People’s Republic of China for inquiries based on the legal name in the article; website, http: //www.moj.gov.cn/, last visit date 27 October 2020.

## Abbreviations

hESC: Human embryonic stem cell
CPL: China Patent Law
GPE: Guidelines for Patent Examination
CPRB: Patent Reexamination Board of CNIPA
CSIPO: The State Intellectual Property Office of CNIPA
CHGC: The China National Human Genome South Research Center

## Footnotes

1 The data were retrieved from the European Patent Office website with patent names and abstracts including keywords “(stem or pluripotent or totipotent or precursor or progenitor) and cell”

2 The data were retrieved from CSIPO with keyword “human embryo”

3 According to article 5 of the CPL, no patent right shall be granted for invention or creation that violates the law or social morality or impairs the public interest; no patent right shall be granted for invention or creation when genetic resources are obtained or utilized in violation of the provisions of laws or administrative regulations and when such genetic resources have been relied on herein

4 Article 25 of the CPL: No patent right shall be granted to the following items: (1) scientific discoveries; (2) rules and methods of intellectual activities; (3) methods for the diagnosis and treatment of diseases; (4) varieties of animals and plants; (5) substances obtained by nuclear transformation; (6) designs mainly for marking the patterns, colors or combination of the two of printed matter. A patent right may be granted in accordance with the provisions of this law with respect to the method of production of the products listed in Item (4) of the preceding paragraph

5 CSIPO patent application CN200780008235.3

6 Decision No. 27204 issued by the Patent Reexamination Board of CNIPA

7 Decision No. 89657 issued by the Patent Reexamination Board of CNIPA

8 Decision No. 26398 issued by the Patent Reexamination Board of CNIPA

9 Decision No. 22325 issued by the Patent Reexamination Board of CNIPA

10 Decision No. 24343 issued by the Patent Reexamination Board of CNIPA

11 Decision No.46359, 42,698 and 115,088 issued by the Patent Reexamination Board of CNIPA
